# Eating disorders risk assessment in men who practice team sports

**DOI:** 10.3389/fnut.2022.957075

**Published:** 2022-09-29

**Authors:** Daniel Baldó Vela, Noelia Bonfanti, Luis Antonio Villarino Marín

**Affiliations:** ^1^Faculty of Nursing, Physiotherapy and Podiatry, Complutense University of Madrid, Madrid, Spain; ^2^Real Madrid University School, European University of Madrid, Madrid, Spain

**Keywords:** eating disorders, men, athletes, sports, team sports, psychology, nutrition

## Abstract

**Introduction:**

Eating disorders (EDs) are characterized by an overconcern about body weight and shape. Men who practice team sports have been systematically excluded from the high-risk eating disorders groups. This exclusion could be challenged based on misinformation about the prevalence of actual eating disorders within these athletes, with the previous evidence showing significant body image concerns among them and the under-diagnosis risk in populations of men.

**Objective:**

To assess eating disorders risk in Spanish adult men who practice team sports.

**Methodology:**

An observational study was conducted with 276 athlete men aged between 18 and 55 years: 60.5% were team sports players and 39.5% were athletes of aesthetic, endurance, and weight-category sports. Data were collected *via* an online form including a general assessment sheet and four validated questionnaires: The Eating Habits Questionnaire for Athletes (CHAD), the Eating Attitudes Test (EAT-40), the Inventory of Eating Disorders (EDI-2), and the Body Shape Questionnaire (BSQ). Data analysis was conducted with the software IBM SPSS 28.0.0.

**Results:**

About 20.36% of team sports players presented a clinical profile compatible with an ED diagnosis. There were no significant differences comparing EDs potential cases between team sports players and athletes playing sports categorized as high EDs risk. There were significant differences when analyzing the existence of EDs between the different groups of age, family pressure, and coach pressure. The *U*-value of the Mann–Whitney test presented significant differences when assessing the influence of BMI over the development of EDs.

**Conclusion:**

Men who practice team sports may also be a high-risk group for the development of EDs. Being younger than 21 years, having a BMI larger than 25 kg/m^2^, and perceiving high-level pressure from the coach and/or family would be risk factors for EDs in men who practice team sports.

## Introduction

Eating disorders (EDs) are a group of mental illnesses characterized by a set of dysfunctional thoughts, actions, and strategies regarding food consumption and/or absorption (1, 2). The main characteristic of these illnesses is the presence of overconcern about body weight and shape. The fear of not getting a stereotypical body can become the everyday focus, reducing the interest in other parts of physical, psychological, and social life. Thus, EDs are a threat to health and wellbeing, leading to physical illness or even death (1–4).

In the case of athletes, the imbalance originated by an ED can worsen due to the practice of exercise without the necessary availability of energy and nutrients (5–9). Furthermore, the development of an ED in these individuals may lead to a strong alteration in their performance, which at the same time brings undesired psychological and social effects (2, 5, 6, 8, 9).

Despite the strong impact of these disorders on athletes, the prevalence of EDs has not been sufficiently analyzed in this population (2, 4, 6, 9–18). Nevertheless, it is known that the risk of EDs is greater in athletes than in the general population. According to the current scientific evidence, the groups with a higher prevalence of EDs are women, adolescents, and athletes competing in gymnastic, aesthetic, endurance, and weight-category sports (2, 4, 5, 10, 13, 14, 16, 19–22).

On the other hand, men have not been considered a high-risk population of EDs. In fact, men account for just 10% of the diagnosed ED cases. Nevertheless, several studies warn about a potential under-diagnosis of EDs in men, and it is estimated that men could represent around 40% of ED cases. The main difficulties when detecting EDs in men when compared to women were as follows (23–28):

- In many cases of EDs in men, overeating and binge eating are the main symptoms, and these behaviors are commonly normalized in them (23, 27, 28).
- In general, men with EDs do not have extremely thin body shapes, and thus their ED could easily pass unnoticed (27, 28).
- A lot of men with some ED are not detected by healthcare professionals and society, as EDs are commonly considered as a group of pathologies that can only affect women. At the same time, the popular association between EDs and women could imply that men with EDs are unconscious or ashamed of their existence, and they do not ask for help (23, 27, 28).
- Etiopathogenesis of EDs is different between men and women. In men, being LGBTQ +, previous existence of obesity, and emotional management skills are more important (28, 29).
- The main symptoms of EDs are different in men: body dissatisfaction is focused on the upper body, obsession with body muscularity is more common than toward thinness, diet deprivation is not frequent, and abusing physical exercise as a method to control body shape and weight is the most common behavior. At the same time, obsession with eating is focused on improving their performance in sports (27, 28).

Due to this evidence, men who practice team sports have been excluded from the high-risk population of EDs (2, 5, 10, 13, 14, 16, 19–21, 23, 30). However, we challenge the veracity of this exclusion and propose its reconsideration founded on the following facts: (a) existing evidence concerning EDs in sports is scarce, particularly when it comes to men and team sports players (2, 6, 12); (b)various studies warn about a potential under-diagnosis of EDs in men (23–28); (c) there is previous evidence of elevated concern regarding body image in team sports players, and previous studies do not find significant differences in the risk of EDs between these and other athletes generally accepted to be at high risk of these illnesses (11, 31, 32); (d) it is not yet known with confidence whether the team acts as a defensive factor or as a risk factor in the development of EDs (14, 33); and (e) stress is an important risk factor for the development of EDs and is higher in the case of men who practice team sports than in the case of women and in the case of athletes from individual modalities (5).

This research raises the following hypothesis: men who practice team sports are a high-risk population for the development of EDs. Therefore, the main aim of this study is to assess the EDs risk in men who practice team sports. The secondary aims are the following:

- To detect symptoms of EDs in men who practice team sports.
- To compare the prevalence of the potential cases of EDs between men who practice team sports and other athlete men who are traditionally considered as a high-risk population to develop some ED.
- To identify the situations with more vulnerability for ED development in men who practice team sports.

## Materials and methods

An observational study was conducted with 276 athlete men: 167 team sports players (from basketball, handball, baseball, football, American football, indoor football, hockey, rugby, volleyball, and water polo) and 109 athletes of the aesthetic (figure gymnastic and skating), endurance (cycling, running, climbing, triathlon, and canoeing), and weight-category sports (rowing, judo, and taekwondo). The inclusion criteria were as follows: being a man; practicing some team, aesthetic, endurance, or weight-category sports; and being federated in Spain. Those who were under 18 or over 55 years old, those who did not grant express consent to their participation, or those who made mistakes when completing the requested documentation were excluded from the sample.

Data collection was carried out between January and July 2021. First, all the Spanish team, aesthetic, endurance, and weight-category sports federations, as well as their main clubs, were contacted by email. Equality, Sports Council, and the four high-performance centers of Spain (Barcelona, León, Madrid, and Granada) were emailed. The purpose of the investigation was explained to them, as well as the criteria that athletes had to meet to participate in the investigation. In addition, the URL to a questionnaire as an online form was sent to them so that they could distribute it among the players that met the criteria.

At the same time, participation was required through direct contacts, through public display of informational advertisements, and through social media.

The self-administered form was available *via* the *Google Forms* platform, and it included a sheet for general assessment: age (years), weight (kg), height (cm), body fat (%), amount of training (hours per week), played sports, sick-leave history (yes or no and reasons), playing position (attacker or defense), role within the team (active, book, or leave), competition level (amateur, semi-professional, or professional), perceived pressure level from the environment (very low, low, high, or very high), and participation in advertisement campaigns (yes or not). Moreover, the online form included the following questionnaires for the detection of EDs:

• Athlete’s Eating Habits Questionnaire (CHAD). Currently, it is the only screening questionnaire that is valid, reliable, and specific for the detection of EDs in the sports field available in the Spanish language (Alfa Cronbach’s = 0.93). A score greater than 100 indicates a potential ED (4).
• Eating Attitudes Test (EAT-40). This test was selected for being one of the questionnaires with the greatest background in the detection of symptoms compatible with anorexia nervosa (Cronbach’s Alpha = 0.93). A score higher than 21 is compatible with the presence of anorexia nervosa (14, 34–37).
• Eating Disorders Inventory (EDI-2). This test was included to evaluate the typical cognitive-behavioral characteristics of anorexia nervosa and bulimia nervosa (Cronbach’s Alpha= 0.90–0.94). A score higher than 105 warns about the potential presence of some ED (4, 5, 14, 34, 36, 38, 39).
• Body Shape Questionnaire (BSQ). This test was specifically included to assess concern and perception of body image. Its reliability is guaranteed by a Cronbach’s Alpha of 0.93–0.98. A score greater than 110 could be indicative of ED (14, 19, 40–43).

In order to reduce the under-diagnosis declared in previous investigations with men (23–28), all four described questionnaires were included to provide a more in-depth evaluation.

The present investigation was approved by the Investigations Commission of the Faculty of Nursing, Physiotherapy, and Podiatry of the Complutense University of Madrid. Moreover, all participants signed an informed consent form and had the opportunity to contact the main researcher to solve doubts about the content or the right way to fill in the evaluation tools. The data collected were anonymized to comply with Spanish Organic Law 3/2018, December 5, on the Protection of Personal Data and Guarantee of Digital Rights.

The data analysis was performed using the statistical software IBM SPSS version 28.0.0. First, the normality of the sample was tested using the Kolmogórov–Smirnov test, which indicated an abnormal distribution in all the variables and sample groups (*p* < 0.05). Consequently, descriptive non-parametric statistics (mean and interquartile range) were calculated in the A group (team sports players), the B group (aesthetic, endurance, and weight-category sports), and the full sample (A and B groups). After that, the prevalence of EDs potential cases was determined in the A and B groups. The prevalence of EDs was determined by considering as an active case any subject scoring above the cut-off point in at least one of the four used questionnaires. Next, the odds ratio was calculated to discern if the EDs risk would be different between team sports players and the commonly considered high-risk group for EDs in athletes.

On the other hand, the χ^2^ test was performed to evaluate the influence of the different variables on the prevalence of EDs in men who practice team sports. For this test, the variables were distributed into groups as follows: age (<21, 21–34, and >34 years), BMI (<24.9 and ≥25 kg/m^2^), body fat (≤20 and >20%), sports modality (American football, basketball, baseball, football, handball, hockey, rugby, indoor football, volleyball, and water polo), competition level (amateur, semi-professional, and professional), playing position (attacker and defense), current role within the team (active, book, and leave), training volume (≤12 and >12 h/week), sick-leave history (yes and no), sick-leave period (days, weeks, months, and years), sick-leave reason (injury, confinement because of COVID19 infection, and incompatibility with life balance), advertisement campaign participation (yes and no), and perceived pressure level from team colleagues, family, friends, coach, and society (very low, low, high, and very high). Finally, the *U*-value of the Mann–Whitney test was made to assess the impact of non-grouped quantitative variables (age, BMI, body fat, and training volume) on the EDs prevalence in men who practice team sports.

## Results

### Participants

Initially, ata were collected in 311 records that met the inclusion criteria. However, after applying the exclusion criteria, the final sample made up to **276 subjects**: 60.5% men who practice team sports (A group) and 39.5% athlete men of the aesthetic, endurance, and weight-category sports (B group). In all cases, the exclusion was due to the detection of multiple participations by the same subject.

### Description of the sample

**FIGURE 1:**
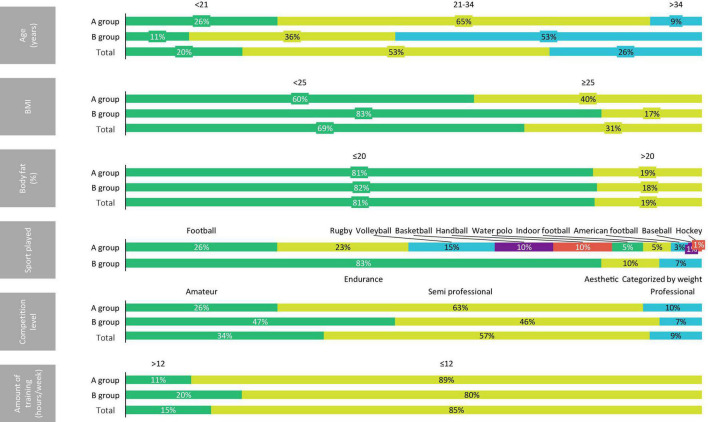
Distribution of the sample according to the age, BMI, body fat, sport played, competition level, and amount of training.

**FIGURE 2:**
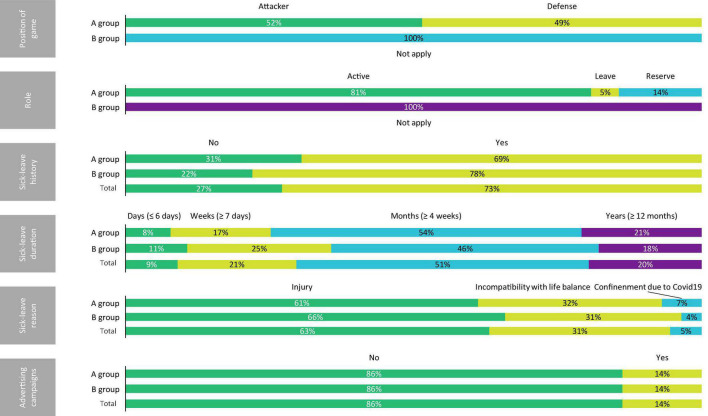
Distribution of the sample according to the position of game, role, sick-leave history, sick-leave duration, sick-leave reason, and participation in advertising campaigns.

**FIGURE 3:**
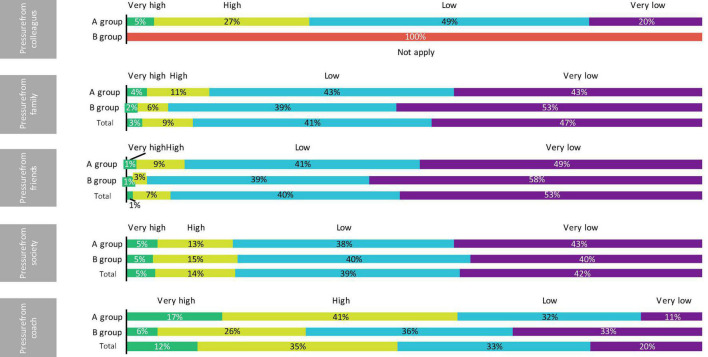
Distribution of the sample according to the pressure perceived from colleagues, family, friends, society, and coach.

Table 1 shows the descriptive statistics (median and interquartile range) of the A group, B group, and global sample. Furthermore, in Figures 1–3, the distribution of the sample may be observed according to the different variables.

**TABLE 1:** Sample’s statistical descriptors: characteristics of Spanish team sports players and athletes playing sports categorized as high risk for EDs (*N* = 276).

	A group				B group				Total sample			
			
	Team sports players				Athletes of the aesthetic, endurance, and weight-category sports				A group and B group			
			
	N	Q1	Median	Q3	N	Q1	Median	Q3	N	Q1	Median	Q3
Age (years)	167	20.00	24.00	30.00	109	25.00	35.00	44.50	276	21.25	27.00	35.000
Weight (kg)	167	73.00	80.00	90.00	109	66.00	71.00	78.00	276	69.25	76.00	85.45
Height (cm)	167	176.00	182.00	188.00	109	172.00	177.00	180.00	276	174.25	180.00	185.00
Body mass index	167	22.50	24.20	26.60	109	21.60	22.80	24.50	276	22.10	23.50	25.55
Body fat (%)	127	11.00	14.00	20.00	88	10.55	14.00	19.88	215	10.70	14.00	20.00
Training volume (hours/week)	167	5.00	8.00	10.00	109	7.00	10.00	12.00	276	6.00	8.00	11.00
CHAD^a^ score	167	53.00	68.00	89.00	109	54.00	70.00	87.50	276	53.00	68.00	88.00
EAT40^b^ score	167	6.00	9.00	13.00	109	6.00	10.00	14.50	276	6.00	9.00	14.00
EDI2^c^ score	167	26.00	33..00	45.00	109	24.00	29.00	42.50	276	25.00	31.50	45.00
BSQ^d^ score	167	41.00	54.00	74.00	109	42.00	52.00	74.00	276	42.00	52.00	73.75

^a^Athlete’s eating habits questionnaire.

^b^Eating attitudes test.

^c^Eating disorders inventory.

^d^Body shape questionnaire determination of the prevalence of EDs and odds ratio in the studied groups.

According to the analysis of the collected data, 20.36% of the A group (team sports players) provided a clinical picture compatible with the existence of one ED (see Table 3). In the B group (athletes of aesthetic, endurance, and weight-category sports), the prevalence of the potential cases of EDs was 20.18% (Tables 3, 4).

Risk estimation through odds ratio did not show statistically significant differences between the A and B groups (OR = 0.98; CI 95% 0.54–1.8).

### Evaluation of the influence of the different variables on the prevalence of eating disorders on team sports players

As shown in Table 2, the χ^2^ test showed statistically significant differences when analyzing the existence of EDs in the different age groups, perceived pressure from family, and perceived pressure from coach (*p* < 0.05). On the contrary, the test did not show statistical significance when assessing the influence of the rest of the variables on the development of EDs (*p* > 0.05).

**TABLE 2:** Results of pearson χ^2^ and U of Mann–Whitney tests.

	Pearson χ ^2^ test			U of Mann-Whitney	
		
	Value	df	Asymptotic significance (bilateral)	Value	Asymptotic significance (bilateral)
Age (years)–EDs	7.76	2	0.02*	1107.50	0.58
Body mass index–EDs	1.96	1	0.16	1671.50	0.02*
Body fat (%)–EDs	2.44	1	0.12	1048.50	0.35
Sport modality–EDs	7.64	10	0.66	–	–
Competition level–EDs	2.98	2	0.22	–	–
Training volume (hours/week)–EDs	1.66	1	0.19	1137.00	0.71
Playing position–EDs	0.93	1	0.33	–	–
Current role–EDs	1.55	2	0.46	–	–
Sick-leave history–EDs	3.34	2	0.06	–	–
Sick-leave reason–EDs	1.21	3	0.75	–	–
Sick-leave duration–EDs	5.91	3	0.11	–	–
Perceived pressure from colleagues–EDs	2.61	3	0.46	–	–
Perceived pressure from family–EDs	11.13	3	0.01**	–	–
Perceived pressure from society–EDs	6.26	3	0.09	–	–
Perceived pressure from friends–EDs	4.62	3	0.20	–	–
Perceived pressure from trainer–EDs	8.23	3	0.04*	–	
Advertising campaigns participation–EDs	3.42	1	0.06	–	–

*It is statistically significant (*p* < 0.05).

**It is statistically significant (*p* < 0.01).

The *U*-test of Mann–Whitney showed significant differences when assessing the impact of body mass index (BMI) on the development of EDs (*p* < 0.05). The study of the impact of the rest of the non-grouped quantitative variables on the EDs existence did not show statistically significant differences (*p* > 0.05; Table 2).

## Discussion

The main aim of this study was to assess the EDs risk in men who practice team sports. The results showed that 20.36% of the studied sample of men who practice team sports had a clinical picture compatible with ED diagnosis. This study did not find significant differences in the EDs risk between the team sports players and the athletes of sports modalities traditionally considered as high-risk for the EDs development. The men who practice team sports with the highest EDs risk would be teenagers, individuals with BMI >25 kg/m^2^, and subjects with a high perceived level of pressure from the coach and/or family.

### Prevalence of eating disorders potential cases

It is important to state that in all used questionnaires, a score above the cut-off point means that there is a clinical picture compatible with an ED diagnosis. All questionnaires are screening tools, and although they alert the presence of EDs, they do not specify the type of ED (5, 14, 34–36, 38, 39, 42).

A fifth of the team sports players (20.36%) and the athletes of aesthetic, endurance, and weight-category sports (20.18%) showed symptoms compatible with a diagnosis of ED.

The prevalence found in the present study when looking for potential EDs cases within the team sports players is similar to the prevalence of 14% reported by Baldó and Bonfanti (11) when analyzing 49 semi-professional basketball, soccer, and rugby players aged between 18 and 35 years. However, this prevalence rate is much higher than the rate found by other studies:

- Teixidor, Ventura and Andrés (30) concluded a 2.4% EDs prevalence when studying 165 men aged 12–56 years who practiced some ball sport in elite categories.
- Martínez et al. (22) observed a 3% prevalence of EDs in 36 handball elite teenager and adult players.
- Samar and Togay (44) noticed a 9% EDs prevalence in a sample of 74 handball players aged 10–49 years.

The lower prevalence observed by Teixidor, Ventura and Andrés (30) could be explained by the methodology used (EAT-26), which could have produced an under-diagnosis that other researchers have already warned about when studying the prevalence of EDs in men (23–28). The EAT-26 has been validated for the recognition of EDs, and it is mainly centered on the preoccupation with thinness and the obsession with diet. Nevertheless, in men with ED, the body concern is not typically focused on slimness, and it is usual for them to control their physique through an obsessive physical workout and not *via* food deprivation (28). What is more, EAT-26 is a questionnaire that has not been validated in athletes, which ignores other researchers’ recommendations in order to avoid the heterogeneity of the prevalence results of EDs in the sports populations (3, 6, 11).

At the same time, the differences in the level of competition of the study sample in the Teixidor, Ventura and Andrés (30) and Martínez et al. (22) investigations with respect to the sample used in the present investigation could also explain the contrast in prevalence between those studies. According to Baldó and Bonfanti (11) and Alonso (10), semi-professional players have higher prevalence rates of EDs than the players of the rest of the competition categories.

On the other hand, with respect to EDs prevalence in athletes who practice aesthetic, endurance, and weight-category sports (all those considered high-risk sports for EDs), the ratio found in the present study is similar to the ratio observed in other investigations with high-risk populations of EDs:

- Martinsen and Sundgot (45) found an EDs prevalence of 25% when they studied a sample of 611 teenager athletes.
- Filaire, Rouvier, Bouget and Pannafieux (46) identified an EDs prevalence of 19% in a sample of 71 athlete women that practiced sports generally considered to be at high risk for the development of EDs.
- Barrientos et al. (47) found an EDs prevalence of 15% in a sample of 169 gym users aged between 15 and 48 years.

It is important to underline the lack of concordance between the results of the four questionnaires used in the present investigation for the EDs detection in both studied groups (Tables 3, 4). The higher sensibility of the CHAD could be justified because it is the only questionnaire specific for athletes that has been validated in male populations. However, the rest of the questionnaires used are focused on ED symptoms that are not common in the majority of ED cases in men (food deprivation, body dissatisfaction oriented to the lower part of the body, and obsessive thinness). These findings show that it would be important to create specific tools to detect EDs in athlete men in order to reduce the under-diagnosis risk alerted in this and other investigations (23–25, 27, 28).

**TABLE 3:** Prevalence of EDs in Spanish team sports players (A group, *N* = 167) and in athletes playing sports categorized as high risk for EDs (B group, *N* = 109).

		1 or more questionnaires	Athlete’s eating habits questionnaire (CHAD)	Eating attitudes test (EAT-40)	Eating disorders inventory (EDI-2)	Body shape questionnaire (BSQ)
Subjects (%) with score >cut-off point	A group	20.36	16.8	7.8	1.8	7.2
	B group	20.18	16.5	14.7	0.9	7.3

**TABLE 4:** Participants diagnosed with a particular EDs risk questionnaire and the prevalence detected with other EDs risk questionnaires among Spanish team sports players (A group, *N* = 167) and athletes playing ED high-risk sports (B group, *N* = 109).

	A group	B group
Participants diagnosed with CHAD^a^ questionnaire	*n* = 28	*n* = 18
% with score > cut-off point in EAT40^b^ questionnaire	32.1%	68.4%
% with score > cut-off point in EDI2^c^ questionnaire	10.7%	5.3%
% with score > cut-off point in BSQ^d^ questionnaire	32.1%	50.0%
Participants diagnosed with EAT40^b^ questionnaire	*n* = 13	*n* = 16
% with score > cut-off point in CHAD^a^ questionnaire	69.2%	81.2%
% with score > cut-off point in EDI2^c^ questionnaire	15.4%	6.2%
% with score > cut-off point in BSQ^d^ questionnaire	46.1%	37.5%
Participants diagnosed with EDI2^c^ questionnaire	*n* = 3	*n* = 1
% with score > cut-off point in CHAD^a^ questionnaire	100%	100%
% with score >cut-off point in EAT40^b^ questionnaire	66.7%	100%
% with score > cut-off point in BSQ^d^ questionnaire	100%	0%
Participants diagnosed with BSQ^d^ questionnaire	*n* = 12	*n* = 8
% with score > cut-off point in CHAD^a^ questionnaire	75.0%	100%
% with score >cut-off point in EAT40^b^ questionnaire	50.0%	75%
% with score >cut-off point in EDI2^c^ questionnaire	25.0%	12.5%

^a^Athlete’s eating habits questionnaire.

^b^Eating attitudes test.

^c^Eating disorders inventory.

^d^Body shape questionnaire.

What is more, it is important to stress that all individuals who scored above the cut-off point in EDI-2 also did it in CHAD. This finding suggests the possibility to avoid EDI-2 as a methodological tool in future research in athlete men without the risk of under-diagnosis of EDs. Since EDI-2 is the longest questionnaire of the four questionnaires used in the present study (91 items), this action could decrease the risk of opt-out and therefore increase participation.

### Prevalence of eating disorders potential cases: Comparison between A and B samples

The estimation of EDs risk through odds ratio did not show significant differences between team sports players and athletes of aesthetic, endurance, and weight-category sports, even though the last group is systematically considered a high-risk population of EDs (2, 4, 5, 13, 14, 16, 19–21, 23, 30). Baldó and Bonfanti (11) reached the same conclusion when comparing the prevalence of EDs potential cases among men who practice team sports and the results offered by other investigations conducted in populations usually considered as high-risk for EDs.

This finding suggests that men who practice team sports should be considered a group at high risk for the development of EDs just like the athletes of aesthetic, endurance, and weight-category sports by weight (2, 4, 5, 13, 14, 16, 19–21, 23, 30).

It is important to mark that the lack of significant differences in EDs risk between team sports players and athletes of aesthetic, endurance, and weight-category sports could be explained by the age difference among the studied samples (Table 1). As it will be described later, being under 21 years would be an important risk factor for the development of EDs in men who practice team sports, and this age group is more represented in the team sports players and athletes of aesthetic, endurance, and weight-category sports.

At the same time, the higher representation of subjects with high or very high perceived pressure from the sports coach in the team sports players with respect to athletes of aesthetic, endurance, and weight-category sports (Figure 3) could also explain the lack of significant differences between the EDs risk in both groups. Nevertheless, this finding could be a sample bias or a characteristic inherent to the team sport practice and, thus, a cause that would explain the high risk of EDs attributed to this population in the present study.

In the last term, it is important to mark that, according to the data presented in Table 3, the number of individuals who scored over the cut-off point in EAT-40 was higher in the athletes of aesthetic, endurance, and weight-category sports than in the team sports players. In the same line, based on the results shown in Table 4, the correlation between CHAD and EAT-40 was higher between athletes of aesthetic, endurance, and weight-category sports. EAT-40 assesses the presence of the typical symptoms of anorexia nervosa (14, 34–37); thus, this finding means that the prevalence of these symptoms would be higher in athletes of aesthetic, endurance, and weight-category sports. Anorexia nervosa has been the most known and studied ED, while others like binge eating disorder and unspecified ED have gone unnoticed. In fact, this finding could explain why athletes of aesthetic, endurance, and weight-category sports have been systematically considered a high-risk group of EDs. At the same time, this finding would justify why team sports players have been excluded from the high-risk population of EDs repeatedly, since in these cases, the typical clinical picture of ED is not attributed to the diagnosis of anorexia nervosa.

### Influence of the different variables on eating disorders risk in male team sports players

Regarding age, the χ2 test showed statistically significant differences when analyzing the existence of EDs between the different age groups. In this sense, considering the higher representation of the subjects under 21 years in the clinical subsample, the highest risk of EDs is linked to this population. Nevertheless, the *U*-value of the Mann–Whitney test did not show significant differences when comparing the median age between the total sample and the clinical subsample, thus the influence of age on EDs risk could be explained by the age group. In this sense, adolescence, that is, the period from 11 to 21 years old, would be the age interval with the highest EDs risk within the studied sample. This finding has already been endorsed by other investigations (3–5, 10, 48, 49) and could be justified by corporality and peer pressure inherent to youth, a fact that would imply a greater concern for reaching the current aesthetic stereotype and, therefore, an increased risk of developing an ED (48, 49).

Subjects were separated into two groups according to their BMI: ≤25 kg/m^2^ and >25 kg/m^2^. Individuals with underweight BMI (<18 kg/m^2^) were not found. χ^2^ test did not show statistically significant differences when analyzing the existence of EDs between the different BMI groups, although the group >25 kg/m^2^ is more represented in the clinical subsample than in the global sample. Also, the *U*-value of the Mann–Whitney test showed significant differences when comparing the median of BMI between the global sample and the clinical subsample. These results suggest that the higher the BMI, the higher the risk of EDs. This finding has already been pointed out by other investigations (11, 50, 51) and could be the consequence of greater body dissatisfaction because of the discrepancies between large bodies and the established aesthetic ideal (14, 40, 43).

On the other hand, the χ^2^ test showed statistical significance when assessing the influence of perceived pressure level from the sports coach on the development of EDs. This finding is supported by previous knowledge (2, 13, 14, 19, 30, 51) and could be justified because the high perceived pressure level from the trainer is usually represented as follows:

- Negative comments about weight and body image, prescription of unhealthy methods for weight loss, and excessive use of the scale as a way of body control. These practices lead the player to create or consolidate the belief that the reasons for his successes and failures are exclusively linked to his body and that he must do everything to adapt it to the requirements of the sport. One of these commonly used practices includes the implementation of dangerous eating behaviors (52–56).
- Motivation based on results. It implies that the player only makes a positive self-evaluation when he gets the awaited results, thoughtless of his progress and efforts. This point, along with the idea of a single body shape as a way to sport success, contributes to the EDs development in athletes (2–5, 10, 13, 14, 19, 20, 49).
- Authoritarianism and unidirectional communication. In both cases, the perceived stress level and, in consequence, felt dissatisfaction are increased. Both feelings are relevant to risk factors for EDs (18, 57–59).
- Low generated self-esteem. The previous points imply important damage to self-esteem and, therefore, a higher need of getting the body shape perceived as a single resource of success and the pressure to get it. This implies a higher risk of EDs (49, 60, 61).

At the same time, the level of pressure perceived from family is also an influencing factor in the EDs development in the case of men who practice team sports. This finding is in line with previous knowledge about the influence of family over the development of EDs in the general population. Evaluation based on the results, critical attitudes from family, and the correlation between success and thinness promotes body dissatisfaction and low self-esteem. As a consequence, athletes could develop highly risky behaviors for the development of EDs in order to “improve” the body as a way to increase results (49, 61, 62).

For the rest of the variables, χ^2^ and the *U*-value of Mann–Whitney tests did not show statistically significant differences when analyzing their impact over the EDs development. However, some appreciations may be made:

- Volleyball players are more represented in the clinical subsample than in the total sample. Nevertheless, the χ^2^ test did not show statistically significant differences, and the previous research does not support this result (14). Since the influence of the rest of the variables has been dismissed, this finding could be justified because the sample size was not enough for a sound analysis.
- There were no statistically significant differences when analyzing the prevalence of EDs potential cases between the subjects with sick-leave history and those who have never been on sick leave. However, the first group is more represented in the clinical subsample than the global sample. This finding supports the results of the investigations of Baldó and Bonfanti (11) and Díaz and Dosil (14) and could be justified due to a higher fear of gaining weight or losing body shape because they have experienced it in their previous sick leave. It is important to stress that the compulsive practice of exercise is the main mechanism of weight control in individual men with EDs (27, 28). The lack of statistical significance could be justified due to the sample size.
- Individuals who had been on sick leave for periods under 7 days were more represented in the clinical subsample than in the global sample. This finding could be justified by body concern and fear of losing body shape, both typical characteristics of subjects with EDs. These symptoms could have made the affected participants restart their sport activity as soon as possible to minimize the consequences of sick leave on their body composition and shape (27, 28). However, the χ^2^ test did not show statistical significance. The lack of statistical significance could be explained due to the scarce representation of individuals who had been on sick-leave periods under 7 days in relation to the rest of the groups of the “sick-leave duration” variable. Therefore, this point needs further investigation.
- Team sports players who participated in the advertisement campaigns were more represented in the clinical subsample than in the total sample, although the χ^2^ test did not get statistical significance. This over-representation in the clinical subsample could be justified by a higher pressure over the body due to the higher body exposition given by the participation in advertising campaigns. At the same time, the use of body edition techniques is common in the advertising field, which increases body dissatisfaction and the wish for the body shape shown in the images (49, 63–65). Body dissatisfaction could be higher when the economic interests of the sports club are linked to the advertising activities because this could imply a higher pressure on the self-body and an increase in perceived stress (18). Also, participating in advertising campaigns could imply more probability of substance abuse to modify body image (66), a behavior linked to the increase in EDs risk (2, 67).

According to what has been described, men who practice team sports, despite being systematically excluded from high-risk groups of EDs, could be an especially vulnerable population for the development of EDs. This fact could be justified by the existence of higher levels of stress associated with competition as a consequence of the fan and media pressure that men team sports have. Added to this is the increase of social pressure on body shape (3, 10, 13, 14), the increasingly tight relationship between sport and thinness (10, 13, 14, 19), and the lower ability that men have to manage stress and anxiety (29, 40).

This study found that men who practice team sports with the highest EDs risk were most teenagers, individuals with BMI >25 kg/m^2^, and those with a high perceived level of pressure from the coach and/or family. Having sick-leave history, a sick-leave duration of under 7 days, and participating in advertising campaigns could also be important risk factors for the development of EDs.

The typical clinical picture of men who practice team sports is not compatible with an anorexia nervosa diagnosis. This fact could justify why these athletes have usually been excluded from the high-risk populations of EDs, since the rest of EDs have gone unnoticed until the current time.

The lack of coherence between the results given by the four validated questionnaires for the detection of EDs (Tables 3, 4) highlights the need to create specific tools for athlete men in order to avoid under-diagnosis. To encourage the participation of the target population and to get larger studied samples, the new methodological tool should be brief. In this sense, EDI-2 could be avoided as a methodological instrument. On the opposite, CHAD is the tool with the highest sensibility to detect EDs potential cases in men who practice team sports of the four questionnaires used in this investigation.

This study highlights the need to modify the current classification of EDs risk groups within the sports field, as well as to develop and implement specific prevention, early detection, and treatment actions in the studied group. In this sense, it seems that special attention should be more focused on younger players, athletes with BMI >25 kg/m^2^, individuals with high perceived levels of pressure from the coach and families, subjects with sick-leave history under 7 days, and players who participate in advertising campaigns.

## Limits and strengths

This investigation is the first study that assesses the EDs risk in men who practice team sports by comparing it with their own control group. This research used a large methodological tool to avoid the under-diagnosis warned in men.

The size of the studied sample did not reach statistical representation in any of the studied groups, and the median of age was different between both groups, requiring further research with larger samples.

## What is already known on this subject? What does this study add?

It is known that men who practice team sports are systematically excluded from the high-risk population when considering the development of EDs. The findings of this study are as follows:

• Men who practice team sports may be a high-risk group for the development of EDs. Therefore, modifying the current classification of EDs risk groups within the sports field is an important need.
• Younger players, athletes with BMI >25 kg/m^2^, individuals with a high perceived level of pressure from the coach and/or family, subjects with sick-leave history under 7 days, and players who participate in advertising campaigns would be the subjects with the highest risk of EDs development within men who practice team sports.
• Anorexia nervosa would be an uncommon ED in men who practice team sports. This finding could explain why these athletes have systematically been excluded from the populations considered high risk for the development of EDs.
• Developing and implementing specific EDs prevention, early detection, and treatment strategies for men who practice team sports is necessary. In the same line, creating specific tools for athlete men to avoid EDs underdiagnosis is required.

## Data availability statement

The original contributions presented in this study are included in the article/supplementary material, further inquiries can be directed to the corresponding author.

## Ethics statement

The studies involving human participants were reviewed and approved by the Investigations Commission of the Faculty of Nursing, Physiotherapy and Podiatry of Complutense University of Madrid. The patients/participants provided their written informed consent to participate in this study.

## Author contributions

DB: conceptualization, methodology, analysis, research, drafting, editing, and reviewing. DB and NB: sampling. LV and NB: supervision. All authors have read and agreed to the publication of this manuscript.
